# Feasibility and stage at diagnosis for children with cancer: a pilot study on population-based data in a middle-income country using the Toronto childhood cancer stage guidelines

**DOI:** 10.3332/ecancer.2024.1795

**Published:** 2024-11-06

**Authors:** Marceli de Oliveira Santos, Paulo Cesar Fernandes de Souza, Fernanda C da Silva de Lima, Nathalie V Balmant, Carolina Motta, Michele Gonçalves da Costa, Rejane de Souza Reis, Gemma Gatta, Beatriz de Camargo

**Affiliations:** 1 Instituto Nacional de Câncer, Rio de Janeiro, RJ 20.230-240, Brazil; 2 Secretaria de Estado de Saúde de Mato Grosso, Cuiabá-MT 78.049-902, Brazil; 3 Instituto Desiderata, Rio de Janeiro, RJ 22.280-020, Brazil; 4 Fundação do Câncer, Rio de Janeiro, RJ 20.231-048, Brazil; 5 Evaluative Epidemiology Unit, Department of Epidemiology and Data Science, Fondazione IRCCS Istituto Nazionale dei Tumori, Milan 20133, Italy; a In memorium; * Same contributions; ** Population-based Cancer Registry of Aracaju, Sergipe, Brazil; Population-based Cancer Registry of Belo Horizonte, Minas Gerais, Brazil; Population-based Cancer Registry of Cuiabá, Mato Grosso, Brazil; Population-based Cancer Registry of Curitiba, Paraná, Brazil.

**Keywords:** childhood cancer, staging, population data, middle-income country

## Abstract

**Background:**

The aim was to conduct a pilot study in a middle-income country testing the use of the Toronto Childhood Cancer Staging System by Population-Based Cancer Registry (PBCR).

**Methods:**

This study involved first the translation of the Australian pediatric cancer staging manual for 16 types of pediatric tumours. Four PBCRs from different regions of Brazil were selected for a pilot study. The study period was from 2005 to 2014, and data were collected from notification sources, including hospitals, pathological laboratories and routine medical records, and staging was done retrospectively.

**Results:**

We identified 1,560 pediatric cancer cases diagnosed between 2005 and 2014. Notably, 94.7% met Tier 1 criteria, and 91.9% met Tier 2 criteria. The PBCR from Curitiba (south region) demonstrated higher staging feasibility (99.3% Tier 1; 96.7% Tier 2) than from Aracaju (northeast) (87.5% Tier 1; 81.3% Tier 2). Most cases had localised or regional disease (77.7%), while 14.3% were metastatic, and 8.0% could not be staged. Osteosarcoma had the highest metastasis rate (50.0%).

**Conclusion:**

Our study demonstrates the feasibility of collecting pediatric cancer stage data from population-based registries in resource-limited settings, advancing our understanding of pediatric cancer outcomes in Brazil.

## Introduction

Childhood cancer represents a small percentage of the cancer burden; thus, population-based data are less known, especially in low- and middle-income countries. Information on the stage at diagnosis is crucial for comparing outcomes across groups over time. However, collecting this information in population-based cancer registries (PBCRs) has become a challenge, as most of them do not collect this data, and the tumour/node/metastasis (TNM) system, the standard method for cancer staging in adults, is inadequate for assessing disease extent and international comparison [1].

The stage at diagnosis is an important factor in guiding the intensity of treatment that is necessary to cure’ childhood cancer. The Toronto Childhood Cancer Stage Guideline has been built to ensure an international staging system to compare stage distribution and survival among different countries and not to substitute the clinical staging used by the institution [1]. The feasibility of applying the Toronto Childhood cancer guidelines was tested in Europe, Australia and in sub-sahara Africa [2–7]. Botta *et al* [8] are currently conducting the Benchista project, which aims to enhance the understanding of disparities in childhood cancer survival across European regions and identify areas requiring improvements. Furthermore, it seeks to encourage the adoption of the Toronto Staging Guidelines (TGs) from PBCRs, both in Europe and beyond, for the most common solid pediatric cancers.

The overall net survival rate in Brazil, when compared to high-income countries, is lower, often attributed to delayed diagnosis as a significant factor [9–11]. The study by Allemani *et al* [10] analysed the 5-year survival of tumours of the central nervous system (CNS), acute lymphoblastic leukemia (ALL) and lymphomas, in children and adolescents, in different countries, including Brazil. The results showed that in Brazil, 5-year survival was below 40% in Brazil and Mexico for CNS tumours, and below 70% for ALL in Brazil, Chile, Colombia, Peru and Thailand. Five-year survival increased by more than 10% in Brazil, Bulgaria, Croatia and Poland [10]. It is noteworthy that the majority of PBCRs have incomplete data on tumour stage in cases of childhood cancer, as evidenced in a literature review conducted by experts, which analysed various aspects of pediatric cancer in Latin America, including diagnostic processes, time to diagnosis, stage at diagnosis, treatments, complications, survival programs, palliative care and end-of-life services [12].

In this context, a gap in population data becomes evident in low- to middle-income countries that could support the notion of more common advanced-stage diagnoses, as the incidence and mortality of pediatric and adolescent cancer in less developed countries vary significantly. This disparity is further exacerbated by the lack of high-quality PBCRs in these nations, resulting in a shortage of reliable information on national incidence and stages at the time of diagnosis for meaningful comparisons [13–15].

Our aim was to conduct a pilot study in a middle-income country testing the use of the Toronto Childhood Cancer Staging System by PBCR. Moreover, to describe and compare stages at diagnosis among PBCR in four different regions in Brazil with European, Australia and Africa studies.

## Material and methods

We first had permission to translate the staging manual done by the Australian group [16, 17]. A personal meeting among pediatric oncologist, cancer registries and epidemiologist was organised in April 2019. We invited several Brazilian PBCR, and four of 4 different regions showed interest to participated (Northeast (Aracaju), Southeast (Belo Horizonte), South (Curitiba) and Central-West (Cuiaba)) for a pilot study. Local training was prepared and done by one of the members of staging manual translation (NB/BDC).

**Figure 1.**
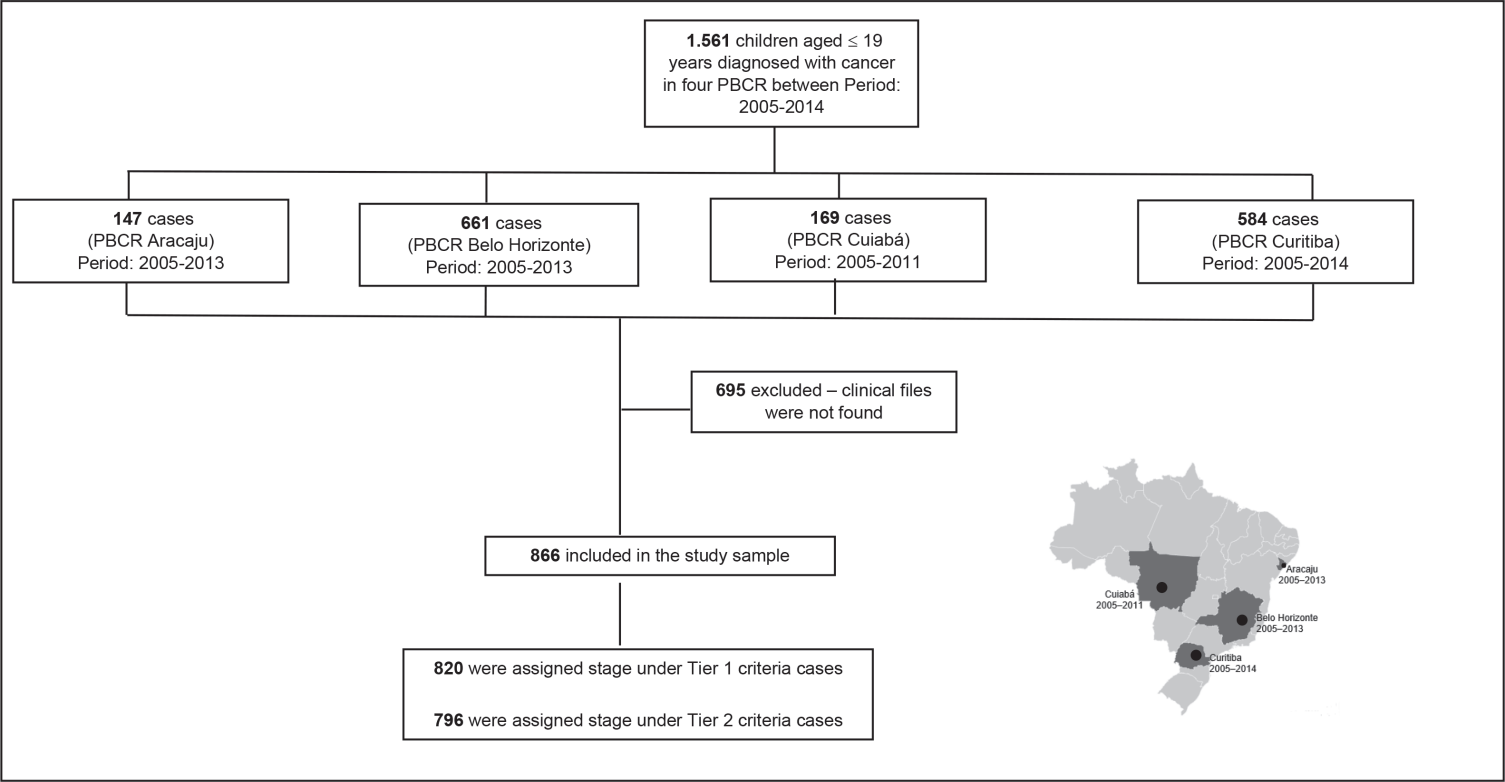
Flow diagram of casuistic selection

The study included different periods of incidence within the years from 2005 to 2014 (Aracaju: 2005–2013; Belo Horizonte 2005–2013; Cuiaba: 2005–2011 and Curitiba 2005–2014). Cases were selected by PBCR – a database with selected cases including the notification sources were done. Notification sources include (specialised hospitals, other hospitals, pathological labs and Mortality Information System) as described in Figure 1. Data collection included demographic variables (sex, residence and age at diagnosis) information on examinations for the diagnosis (microscopic or clinical) cancer site and morphology codified by the ICD-O3, according to the TG, Information was extracted from routine medical records and pathological reports. The staging was done retrospectively for all cases according to Toronto Guidelines that include tier 1 and tier 2 [1] (Supplementary Table).

## Results

### Feasibility

1,560 cases younger than 19 years were identified in the four PBCR during the period between 2005 and 2014. It was not possible to retrieve medical records for 695 cases – most of them in non-specialised departments; therefore, it was possible to perform stage in 866 cases (Table 1). The median collection time was 13 minutes and 42 seconds.

Of the 866 cases, 820 (94.7%) had sufficient information in the medical records possible to apply the stage according to Tier 1 criteria and 796 (91.9%) according to Tier 2 criteria. The PBCR of Curitiba was more feasibility in staging all tumours (99.3 Tier 1; 96.7 Tier 2), and Aracaju had a lower percentage (87.5 Tier 1; 81.3 Tier 2) (Table 2).

For the haematologic tumours, the acute lymphoblastic leukaemia cases stage was reconstructed slightly more (Tier 1 96%, tier 2 91%). Among solid tumours, neuroblastoma and germ cell tumour of testis and ovary stage at diagnosis were totally reconstructed according to Tier 1 and 2 (Table 2), 673 (77.7%) of cases in the study had localised or regional disease at diagnosis, 124 (14.3%) were diagnosed with metastatic cancer and the remaining 69 (8.0%) cases were not possible to perform staging (Table 3). Stage distribution by tumour can be seen in Table 3. Osteosarcoma has the most frequency of advanced disease with 45.0% of new cases diagnosed with metastases at diagnosis (Table 3). On the contrary, medulloblastoma (5%), ependymoma (17%), ovarian germ cell tumour (20%) and rhabdomyosarcoma (21%) were the solid tumours with the lowest percentages of the metastatic stage at diagnosis. Important to note that also in Brazil, no metastatic retinoblastoma was reported. Regarding to neuroblastoma and Wilms tumour comparison with the Australian and European groups can be seen in Table 4. Both with a metastatic lesions higher in Brazilian children versus the Australian and European ones.

## Discussion

The stage of disease represented a major prognostic variable. The type of stage varies among pediatric oncology institutions according to which treatment protocol is used. It is extremely necessary to have a uniform tool to compare international staging and survival at the population level. There are none population-based study regarding stage at diagnosis for childhood cancer in Brazil. This pilot study demonstrates that the Toronto guidelines is feasible in a middle-income country as Brazil. Completeness of data was obtained by medical records in 94.7% Tier 1 and 91.9% Tier 2 of patients with slight differences between PBCR in different geographic regions in Brazil. The study provides also a comparison with the Australian and the European PBCRs showing minor differences. At the time of this study, the European study only provided staging information for neuroblastoma and Wilms’ tumours.

It is important to highlight that notably, published data regarding the stages and survival of pediatric tumours primarily stem from clinical trials, exhibiting variations in selection criteria, in contrast to PBCR data [18, 19]. Given this, the findings of our study are nationally representative due to the standardization of stage information at the time of diagnosis through the utilization of the Toronto consensus, as well as the potential for data comparability across countries.

Diagnosis delay in childhood cancer is one of the predominant causes of disseminated malignancies [20]. It is described in low-middle-income countries that advanced disease is more frequent [21]. In the early 80´s, advanced disease was a major issue in Brazil and professional groups approached medical schools with lectures and local papers. Lay people were reached through newspaper, television, etc. and significant improvement has been noticed [22]. Early diagnosis of cancer is important to allow the opportunity for effective treatment, with fewer side effects and improved survival. Early diagnosis program has been done in Brazil being the biggest program supported by Ronald McDonald Institute since 2008 and significant improvement has been seen in some regions [23]. Several campaigns for early diagnosis of retinoblastoma were implanted [24]. Actually, in our study, we cannot see metastatic retinoblastoma, which was the case in the early 1980’ years.

Surprisingly we found a similar proportion of localised and advanced disease in the majority of tumours comparing with European and Australian data [3, 5]. Interestingly, a study conducted in three African countries demonstrated the feasibility of assigning stage at diagnosis in over 80% of cases based on the Toronto guidelines. However, a large proportion of half of the cases (52%) presented with advanced disease stage. It is worth noting that stage information at the time of diagnosis emerged as a significant predictive variable, as cases with an advanced stage for each of the three analysed tumours (non-Hodgkin lymphoma, retinoblastoma or Wilms tumour) exhibited poorer survival rates [7]. This suggests difficulties in accessing to specialised centers in the African region studied.

In our study, children with ALL were classified as CNS1 in 89% similarly as the Australian data (91%). On the contrary for acute myeloid leukemia (AML), CNS-staged children were higher in Brazil (81%) as in Australia (66%). The extent of CNS involvement can be difficult to know by registrars and CNS involvement is more frequent in the AML. However, delay diagnosis could be not higher than in high-income countries and was not associated with morbidity and early mortality in children with acute leukemia when treated in a specialised pediatric oncology unit in Northeast Brazil [9]. Access to specialised centers and family education may affect the early diagnosis. In a recent 5-year net survival study from Concord 3, the survival rates for all leukemia combined in the 0–14 years age group ranged from 48% (95% CI 26–70) in India to 91% (95% CI 84–98) in Puerto Rico. Among adolescents (15–19 years), survival varied from 24% (95% CI 11–38) in Colombia to 85% (95% CI 75–95) in Denmark. Survival was higher in the 0–14 age group compared to the 15–19 age group. Specifically, in Brazil, the 5-year net survival rates for all combined leukemia, as well as for the subtypes of ALL and AML, were 67.3% (95% CI 59–75), 72.8% (95% CI 64–81) and 42.9% (95% CI 22–63), respectively [25]. Gatta *et al* [26] found that in high-income European countries, more than 80% of children diagnosed with ALL manage to survive for 5 years after receiving the diagnosis.

The Brazilian Renal tumour group has shown improvement of survival but is still inferior to high-income countries. The mean tumour volume at diagnosis among Brazilian children was not higher than in other countries. Unfortunately, this population represented only 10% of the estimated number of cases. Toxicity related to treatment was higher [27]. It is well known that access to centralised expertise for radiology and pathology review can influence stage distributions and treatment. It is the aim of the next Brazilian group participation on the UMBRELLA Renal Tumour Study [28].

Significant differences of localised and advanced disease were seen in patients with osteosarcoma. We observed a higher proportion to children with metastases than in Australia data. In a study of patients treated in a Brazilian cooperative group, metastases at diagnosis were present in 21% of patients reflecting advanced disease at diagnosis. The time from onset of symptoms to diagnosis did not correlate with the presence of metastases, tumour size or survival suggesting that the stage of disease at presentation depends more on biological aspects of the tumour than late diagnosis [29]. This data confirms that is necessary biological studies in patients with osteosarcoma in Brazil, as the TG-2 suggested. Actually, Gupta *et al* [1] suggested to collect other clinical variables, than stage at diagnosis, such as the so called not stage prognosticators.

Regarding the application of the TGs, our results have revealed a significant proportion of cases with an unknown stage, indicating a lack of comprehensive information in medical records. This gap may be attributed to the need for enhancing the quality of information recorded in patient charts or emphasising closer collaboration with pediatric oncologists to improve the clinical data collection process. Furthermore, in a recent study conducted by Liu *et al* [30], it was demonstrated that the feasibility of applying these guidelines in Sub-Saharan African countries resulted in staging being attainable in 71% of cases, with variations ranging from 53% to 83% for specific cancer types. This level of accuracy is closely approximated that observed in high-income countries, particularly in the context of solid tumours. This finding represents a significant stride toward understanding the challenges posed by pediatric cancer in low- and middle-income countries [30].

Another study on the feasibility of implementing the Toronto guidelines in seven pediatric oncology units in Sub-Saharan Africa from 2017 to 2019 revealed that it was possible to assign the stage at the time of diagnosis in 89% (1,772) of eligible cases for 11 types of cancer, except for leukemia and CNS tumours [31]. The lack of information in medical records prevented it at Tier 2, unlike our results that demonstrated the possibility of staging cases in both Tier 1 and Tier 2. Additionally, the percentage of metastatic cases in our study was 14.3% and 77.7% [31].

Youlden *et al* [32] reported that the distribution of stages has remained stable for most types of childhood cancer in Australia over the past two decades, with the exception of retinoblastoma and hepatoblastoma. Furthermore, significant improvements in 5-year survival rates were observed, particularly among recently diagnosed children, including those with advanced solid tumours.

The World Health Organization [33] launched the Global Initiative for Childhood Cancer in 2018. The purpose of this initiative is to provide support to governments in establishing and sustaining high-quality national childhood cancer treatment programs. The established global objective is to achieve a minimum survival rate of 60% for children and adolescents aged 0 to 19 with cancer by the year 2030. To effectively monitor and evaluate the adopted actions, the need for a high-quality information system and well-structured surveillance of childhood cancer through PBCRs is imperative [34].

This is the first population-based data with a standard staging system and despite the small numbers of cases stage distribution is comparable to other countries among most of the diseases. Stage at diagnosis influences overall and event-free survival, cost of treatment and late effects. Survival rates in Brazil are inferior comparing with high-income countries and advanced disease may not be the only reason [10, 25]. Treatment in a specialised center with a multidisciplinary team and the development of a network of hospitals/departments, is extremely recommend improving survival.

## Strengths and limitations

The strengths of our study included the use of PBCR data and the comparability of disease extent estimates at the population level.

The main limitations of this study include its retrospective nature and the small number of cases, limited to capital cities where PBCRs are available. Part of the data collection occurred during the COVID-19 pandemic, which led to the unavailability of several patient records. Another significant limitation is the lack of follow-up data, preventing the calculation of overall survival. Additionally, 695 cases were excluded due to missing medical records, which may affect the comprehensiveness of the findings. The low incidence of certain tumour types in specific regions could also introduce bias, limiting the generalizability of the results.

It was very important for education among cancer registries and despite the small number of cases; it suggests improvement in reconstructing stage disease in our country.

## Conclusion

Our results demonstrate that it is feasible to collect information on the stage at diagnosis of children diagnosed with cancer from PBCRs, both at Tier 1 and Tier 2, even in resource-limited settings. This represents a significant advancement in the pursuit of a deeper understanding of adverse outcomes in pediatric cancer in Brazil.

## Conflicts of interest

The authors declare no conflicts of interest.

## Figures and Tables

**Table 1.** Characteristics of the study cohort.

Tumor type	Study cohort^a^		Cases included in study		Gender ratio (boys/girls)	Median age at diagnosis (months)
	*N*	*n*		%		
ALL	396	264		66.7	1.0	80
AML	137	73		53.3	1.5	101
Hodgkin lymphoma	191	92		48.2	1.5	185
Non-Hodgkin lymphoma	179	83		46.4	2.3	143
Neuroblastoma	72	46		63.9	1.1	28
Wilms tumor	70	49		70.0	1.7	28
Rhabdommyosarcoma	61	34		55.7	1.7	86
Non-rhadomyosarcoma soft tissue sarcoma	64	20		31.3	1.0	181
Osteosarcoma	85	40		47.1	0.9	158
Ewing sarcoma	56	42		75.0	1.1	162
Retinoblastoma	38	20		52.6	1.9	13
Hepatoblastoma	20	11		55.0	0.4	24
Testicular germ cell tumor	46	26		56.5	-	213
Ovarian germ cell tumor	26	15		57.7	-	192
Medulloblastoma	86	39		45.3	0.9	84
Ependymoma	34	12		35.3	0.1	113
Total	1,561	866		55.5	1.2	103

a All cases of PBCR of the study

**Table 2.** Cases of childhood cancer could be staged according to Tier 1 and Tier 2 criteria of the Toronto guidelines by Brazilian registry.

Tumor type	Tier 1		Tier 2		Tier 1		Tier 2		Tier 1		Tier 2		Tier 1		Tier 2		Tier 1		Tier 2		Total
	*n*	%	*n*	%	*n*	%	*n*	%	*n*	%	*n*	%	*n*	%	*n*	%	*n*	%	*n*	%	*n*
	Aracaju				Belo Horizonte				Curitiba				Cuiabá				All				
ALL	22	88.0	19	76.0	52	92.9	51	91.1	128	99.2	121	93.8	50	92.6	50	92.6	252	95.5	241	91.3	264
AML	2	100.0	2	100.0	17	73.9	17	73.9	38	100.0	38	100.0	9	90.0	9	90.0	66	90.4	66	90.4	73
Hodgkin lymphoma	5	71.4	5	71.4	22	91.7	22	91.7	43	95.6	43	95.6	9	81.8	9	81.8	78	89.7	78	89.7	87
Non-Hodgkin lymphoma	8	100.0	8	100.0	30	85.7	30	85.7	32	100.0	31	96.9	11	84.6	10	76.9	81	92.0	79	89.8	88
Neuroblastoma	5	100.0	5	100.0	13	100.0	13	100.0	24	100.0	24	100.0	4	100.0	4	100.0	46	100.0	46	100.0	46
Wilms tumor	5	100.0	5	100.0	12	92.3	12	92.3	23	100.0	23	100.0	8	100.0	7	87.5	48	98.0	47	95.9	49
Rhabdommyosarcoma	4	100.0	4	100.0	6	85.7	6	85.7	17	100.0	17	100.0	5	71.4	5	71.4	32	91.4	32	91.4	35
Non-rhadomyosarcoma soft tissue sarcoma	0	0.0	0	0.0	5	100.0	4	80.0	10	100.0	9	90.0	4	100.0	3	75.0	19	95.0	16	80.0	20
Osteosarcoma	0	0.0	0	0.0	11	100.0	11	100.0	23	100.0	23	100.0	5	83.3	5	83.3	39	97.5	39	97.5	40
Ewing sarcoma	1	50.0	1	50.0	12	100.0	12	100.0	22	100.0	22	100.0	4	66.7	4	66.7	39	92.9	39	92.9	42
Retinoblastoma	1	100.0	0	0.0	9	100.0	8	88.9	10	100.0	10	100.0	0	0.0	0	0.0	20	100.0	18	90.0	20
Hepatoblastoma	1	50.0	1	50.0	3	75.0	3	75.0	3	100.0	3	100.0	1	100.0	1	100.0	8	80.0	8	80.0	10
Testicular germ cell tumor	0	0.0	0	0.0	6	100.0	6	100.0	17	100.0	17	100.0	3	100.0	3	100.0	26	100.0	26	100.0	26
Ovarian germ cell tumor	1	100.0	1	100.0	6	100.0	6	100.0	3	100.0	3	100.0	5	100.0	5	100.0	15	100.0	15	100.0	15
Medulloblastoma	1	100.0	1	100.0	12	100.0	11	91.7	21	100.0	19	90.5	5	100.0	5	100.0	39	100.0	36	92.3	39
Ependymoma	0	0.0	0	0.0	0	0.0	0	0.0	10	100.0	10	100.0	2	100.0	0	0.0	12	100.0	10	83.3	12
Total	56	87.5	52	81.3	216	91.5	212	89.8	424	99.3	413	96.7	125	89.9	120	86.3	820	94.7	796	91.9	866

**Table 3.** Comparisons of stage distribution between Australia and Brazil.

Tumor type	Staging (Tier 2)	Australia^1^		Brazil (current study)	
		*n*	%	*n*	%
ALL	CNS 1	1.337	91.0	235	89.0
	CNS 2	101	6.9	3	1.1
	CNS 3	31	2.1	3	1.1
	X	-		23	8.7
AML	CNS -	158	65.6	59	80.8
	CNS +	83	34.4	7	9.6
	X	-		7	9.6
Hodgkin lymphoma	IA/B	31	16.2	17	19.5
	IIA/B	67	35.1	32	36.8
	IIIA/B	38	19.9	17	19.5
	IVA/B	55	28.8	13	14.9
	X	-		8	9.2
Non-Hodgkin lymphoma	I	36	12.0	17	19.3
	II	29	9.6	19	21.6
	III	169	56.1	29	33.0
	IV	67	22.3	14	15.9
	X	-		9	10.2
Rhabdommyosarcoma	TNM 1	52	32.7	7	20.6
	TNM 2	21	13.2	5	14.7
	TNM 3	50	31.5	13	38.2
	TNM 4	36	22.6	7	20.6
	X	-		2	5.9
Non-rhadomyosarcoma soft tissue sarcoma	TNM 1	58	46.8	5	25.0
	TNM 2	14	11.3	1	5.0
	TNM 3	26	21.0	4	20.0
	TNM 4	26	21.00	6	30.0
	X	-		4	20.0
Osteosarcoma	L	59	73.8	21	52.5
	M	21	26.3	18	45.0
	X	-		1	2.5
Ewing sarcoma	L	73	67.0	26	61.9
	M	36	33.0	13	31.0
	X	-		3	7.1
Retinoblastoma	0	45	30.4	10	50.0
	1	99	66.9	7	35.0
	2	*	*	1	5.0
	3	*	*	0	0.0
	4	*	*	0	0.0
	X	-		2	10.0
Hepatoblastoma	L	53	66.3	5	50.0
	M	27	33.8	3	30.0
	X	-		2	20.0
Testicular germ cell tumor	I	25	80.7	16	61.5
	II	*	*	2	7.7
	III	*	*	8	30.8
	X			0	0.0
Ovarian germ cell tumor	I	21	50.0	8	53.3
	II	*	*	3	20.0
	III	13	31.0	1	6.7
	IV	*	*	3	20.0
	X	-		0	0.0
Medulloblastoma	M0	179	68.9	28	71.8
	M1	5	1.9	0	0.0
	M2	19	7.3	1	2.6
	M3	58	22.2	5	12.8
	M4	0	0	2	5.1
	X	-		3	7.7
Ependymoma	M0	92	90.2	6	50.0
	M1	*	*	0	0.0
	M2	*	*	0	0.0
	M3	6	5.9	2	16.7
	M4	*	*	2	16.7
	X	-		2	16.7

* Result withheld (cell count <5 or insufficient data)

1 Youlden *et al* [3, 4]

**Table 4.** Neuroblastoma and Wilms tumours, comparisons of stage distribution between Australia, European and Brazilian registries.

Tumor type	Staging (Tier 2)	Australia^1^		European^2^		Brazil (current study)	
Neuroblastoma	L1	98	25.6	166	26.7	14	30.4
	L2	67	17.5	134	21.6	8	17.4
	M	24	6.3	67	10.8	14	30.4
	MS	194	50.7	237	38.2	10	21.7
	X	-		17	2.7	0	0.0
Wilms tumor	I/y-I	69	28.6	196	39.7	25	51.0
	II/y-II	55	22.8	103	20.9	5	10.2
	III/y-III	75	31.1	93	18.8	8	16.3
	IV/y-IV	42	17.4	63	12.8	9	18.4
	X	-		39	7.9	2	4.1

Result withheld (cell count<5 or insufficient data)

1 Youlden *et al* [3, 4]

2 Gatta *et al* [5]

## Supplementary information

**Supplementary Table 1.** Summary adapted of the Toronto guidelines for the staging of pediatric cancer.

Diagnostic group	Staging system (Low resource level 1)	Staging system (High resource level 2)
ALL	CNS-, CNS+, Unknown (X)	CNS1, CNS2, CNS3, Unknown (X)
AML	CNS-, CNS+, Unknown (X)	CNS-, CNS+, Unknown (X)
Hodgkin lymphoma	Stage IA/B, IIA/B, IIIA/B, IVA/B, Unknown (X)	Stage IA/B, IIA/B, IIIA/B, IVA/B, Unknown (X)
Non-Hodgkin lymphoma	Limited, Advanced, Unknown (X)	St. Jude/Murphy I, II, III, IV, Unknown (X)
Neuroblastoma	Localized, Loco-regional, Metastatic, Unknown (X)	INRGSS: L1, L2, M, MS, Unknown (X)
Wilms tumor	Localized (L), Metastatic (M), Unknown (X)	Stages I, II, III, IV, Unknown (X)
Wilms tumor	Localized (L), Metastatic (M), Unknown (X)	Stages y-I, y-II, y-III, y-IV, Unknown (X)
Rhabdomyosarcoma	Localized (L), Metastatic (M), Unknown (X)	Modified TNM: Stages I, II, III, IV, Unknown (X)
Non-rhabdomyosarcoma soft tissue sarcomas	Localized (L), Metastatic (M), Unknown (X)	Modified TNM: Stages I, II, III, IV, Unknown (X)
Osteosarcoma	Localized (L), Metastatic (M), Unknown (X)	Localized (L), Metastatic (M), Unknown (X)
Ewing sarcoma	Localized (L), Metastatic (M), Unknown (X)	Localized (L), Metastatic (M), Unknown (X)
Retinoblastoma	Localized (L), Regional (R), Metastatic (M), Unknown (X)	IRSS Stages 0, I, II, III, IV, Unknown (X)
Hepatoblastoma	Localized (L), Metastatic (M), Unknown (X)	Localized (L), Metastatic (M), Unknown (X)
Medulloblastoma and other embryonal CNS tumors	M0, M1-M4, Unknown (X)	M0, M1, M2, M3, M4, Unknown (X)
Testicular cancer	Localized (L), Regional (R), Metastatic (M), Unknown (X)	Modified TNM: Stages I, II, III, Unknown (X)
Ovarian cancer	Localized (L), Regional (R), Metastatic (M), Unknown (X)	FIGO: Stages I, II, III, IV, Unknown (X)
Ependymoma	M0, M1-M4, Unknown (X)	M0, M1, M2, M3, M4, Unknown (X)

Source: Gupta *et al* [1]
